# Neonatal IL-4 and IL-5 signaling drives long-term inflammation after early-life allergen exposure in a mouse model of allergic sensitization

**DOI:** 10.3389/fimmu.2026.1858193

**Published:** 2026-10-02

**Authors:** Brian W. P. Seymour

**Affiliations:** DeBusk College of Osteopathic Medicine, Lincoln Memorial University, Orange Park, FL, United States

**Keywords:** aeroallergens, allergic sensitization, early-life exposure, eosinophilia, IgE, IL-4, IL-5, immune tolerance

## Abstract

**Background:**

Early-life exposure to allergens is a critical window that shapes immune development. Epidemiological and experimental evidence suggests that neonatal cytokine responses, particularly interleukin (IL)-4 and IL-5, drive type 2 immunity and increase the risk of allergic sensitization and asthma in adulthood. However, whether blocking these pathways in neonates can alter long-term susceptibility remains unclear.

**Methods:**

Neonatal BALB/c mice were exposed to 1% aerosolized ovalbumin (OVA) for 20 min, whereas controls received PBS. A subset of OVA-exposed neonates received 0.5 mg anti-IL-4 antibody at day 0. At adulthood (8 weeks), all groups were reexposed to aerosolized OVA. Blood and lung samples were collected for analysis of eosinophils, OVA-specific IgE, and cytokine production. *In vitro* restimulation of lung cells was performed for type 2 cytokines (specifically IL4, IL5, and IL13) and IFN-γ profiling.

**Results:**

Mice exposed to aerosolized OVA as neonates exhibited elevated IL-4, IL-5, and IL-13, with increased eosinophilia and OVA-specific IgE, consistent with a type 2-biased response. PBS control neonatal mice challenged only in adulthood with aerosolized OVA showed no such sensitization. Strikingly, anti-IL-4 treatment at 2 days old prevented the dysregulated allergic sensitization and restored a balanced immune response, even upon OVA reexposure in adulthood. Anti-IL-4-treated neonates remained capable of mounting normal Th2 responses comparable with those observed in non-sensitized adult mice.

**Conclusion:**

Neonatal IL-4 and IL-5 signaling critically shapes long-term eosinophilic allergic sensitization. Early blockade of IL-4 prevents lifelong allergen-specific dysregulation, highlighting potential therapeutic benefits of cytokine-targeted interventions in infancy for the prevention of allergic diseases.

## Introduction

Pulmonary ventilation, which allows a large volume of air to enter the lungs for the process of cellular respiration, continuously exposes the respiratory tract to a vast array of environmental antigens (1, 2). Although penetration of environmental substances into the deep lungs is largely limited due to the protective epithelial barriers, inhaled antigens can access immune cells within the respiratory mucosa and initiate immune responses (3, 4). Under normal circumstances, the immune system must distinguish harmful pathogens from harmless environmental antigens and maintain tolerance to these innocuous and ubiquitous substances encountered through the airways. Failure of these regulatory mechanisms can result in inappropriate immune activation and the development of allergic airway diseases (4–6).

Allergic diseases are characterized by type 2 immune responses involving T helper 2 (Th2) cells and the production of cytokines such as interleukin (IL)-4, IL-5, and IL-13. These cytokines contribute to the development and maintenance of allergic inflammation through the effects on lymphocyte differentiation, immunoglobulin E (IgE) production, eosinophil recruitment, and airway remodeling (5–8). Among these mediators, IL-4 is widely recognized as a key regulator of Th2 differentiation (9). Signaling through the IL-4 receptor activates STAT6 and promotes expression and enhancement of the transcription factor GATA3, facilitating the development of Th2 immune responses (10–12). IL-4 and IL-13 also contribute to immunoglobulin class switching toward IgE production, a hallmark of allergic sensitization (13–15).

IL-5 is strongly associated with eosinophilic inflammation. It supports eosinophil maturation, differentiation, recruitment, and survival, thereby contributing to the persistence of allergic inflammation (16–20). Elevated IL-5 production and eosinophilia are frequently observed in allergic airway diseases, particularly severe eosinophilic asthma (21). Together, IL-4 and IL-5 represent important components of the type 2 inflammatory pathway and have become major therapeutic targets in allergic diseases (21, 22).

Previous studies using murine models have demonstrated that exposure of adult mice to aerosolized ovalbumin (OVA) can induce antigen-specific immune unresponsiveness (23–25). Exposure of adult mice to inhaled OVA exhibit reduced OVA-specific IgE responses and diminished Th2 reactivity following subsequent immunogenic challenge (intraperitoneal (IP) injection of OVA suspended in aluminum hydroxide). These findings suggest that inhaled antigens can induce a state of immune tolerance under certain conditions. However, the mechanisms governing tolerance induction may differ substantially during early-life immune development.

The neonatal immune system undergoes rapid maturation and exhibits unique functional characteristics compared with adulthood (6). Early-life environmental exposures can influence immune programming and may have long-lasting consequences for susceptibility to allergic diseases. While inhalation tolerance has been extensively studied in adult animals (25), less is known about how inhaled allergen exposure during the neonatal period affects long-term immune outcomes. In particular, the contribution of IL-4- and IL-5-dependent pathways to the balance between allergic sensitization and tolerance during this critical developmental window remains incompletely understood.

For the purposes of this study, allergic sensitization refers to the development of antigen-specific Th2 responses, IgE production, and eosinophilic inflammation following allergen exposure. In contrast, inhalation tolerance refers to the suppression or attenuation of immune responses following subsequent exposure to the same inhaled antigen. Understanding the factors that determine whether early-life antigen exposure promotes sensitization or tolerance is important for identifying mechanisms underlying allergic disease susceptibility.

Therefore, the objective of the present study was to determine whether neonatal exposure to aerosolized OVA influences long-term susceptibility to allergic sensitization and to investigate the contribution of IL-4 and IL-5 signaling pathways to these responses. We hypothesized that neonatal allergen exposure promotes persistent allergic sensitization rather than tolerance and that IL-4- and IL-5-dependent signaling contributes to the establishment of this long-term allergic phenotype.

## Materials and methods

### Experimental design overview

Three related experimental protocols were used to investigate the effects of neonatal allergen exposure on the development of allergic sensitization and inhalation tolerance. The first protocol namely, timeline A evaluated the effect of age at initial aerosolized ovalbumin (OVA) exposure on subsequent allergic (immunogenic) responses in adulthood. The second protocol namely, timeline B examined whether neonatal exposure altered the ability of mice to develop inhalation tolerance following repeated aerosolized OVA exposures in adulthood. The third protocol namely, timeline C investigated whether a single neonatal aerosol exposure was sufficient to alter tolerance development and whether blockade of IL-4 signaling during neonatal exposure modified these outcomes. Detailed timelines for each protocol are provided in the corresponding figure schematics.

The timing of sample collection was based on our previously established kinetic analyses of the antigen-specific immune response in this experimental model. Serum samples for the quantification of OVA-specific IgE by ELISA were collected 7 days after the final antigen exposure, as this time point consistently corresponds to the peak IgE response. For cytokine analyses, samples were collected 3–4 days after the final antigen exposure, based on kinetic studies demonstrating maximal cytokine production at this interval. Thus, the collection time points for each immunological endpoint were selected to capture the peak biological response, thereby maximizing the sensitivity and consistency of the analyses.

### Animals

BALB/cAnN mice were sourced from Simonsen Laboratories in Gilroy, CA, and Taconic in Germantown, NY. They were kept under pathogen-free conditions at the DNAX Research Institute animal facility. All experiments began with mice whose age ranged from 2 days to 8 weeks. Experiments were performed using both male and female mice.

### Allocation of animals to experimental groups

Mice from multiple litters were assigned to experimental groups according to age and treatment protocol. Whenever possible, littermates were distributed among treatment groups to minimize litter-specific effects. Animals were allocated before initiation of experimental procedures and were housed under identical environmental conditions throughout the study.

### Rational for exposure ages

The selected exposure ages were chosen to evaluate immune responses across distinct developmental stages. Neonatal mice (2–7 days of age) were studied to assess immune programming during the earliest period of postnatal immune maturation. Weanling mice (13–18 days of age) were included in the study to examine the transition between neonatal and more mature immune responses. Adult mice (>5 weeks of age) served as the reference group because inhalation tolerance following aerosolized OVA exposure has been previously characterized in mature animals (23).

### Aerosol and intraperitoneal antigen exposure

Aerosolization of OVA or PBS was carried out for 20 min using a Passport aerosol compressor (Invacare Corporation, Elyria, OH) attached to a 3 cubic foot chamber. The aerosol mixture contained 1% OVA in PBS. Control groups included mice exposed to aerosolized PBS and subsequently challenged IP with 10 μg OVA (grade V, catalog #A-5503, Sigma, St. Louis, MO) in 2 mg aluminum hydroxide gel (OVA/AL IP), followed by aerosolized OVA. Another control group, which represents the maximal immunogenic stimulation, received two IP injections of 10 μg OVA/AL IP in adulthood before aerosolized OVA exposure. Blood was collected from tail veins and serum stored at −70 °C for antibody assays.

### In vivo antibody treatment

To evaluate the contribution of IL-4 signaling during neonatal allergen exposure, neonatal mice received a single IP injection of 0.5 mg of anti-IL4 monoclonal antibody (11B11) at the time of aerosolized OVA exposure. Control mice received an equivalent dose of the isotype control antibody, which was GL113. This antibody has been shown to have no immunogenic effect on this mouse model of allergen sensitization (23). Although IL-5 production was measured as an outcome variable in lung cell culture supernatants, direct experimental manipulation of IL-5 signaling was not performed in this study. Functional studies were limited to blockade of IL-4 using 11B11.

### Quantification of eosinophils in the peripheral blood

Eosinophils were counted by diluting heparinized blood in Discombe’s fluid as previously described (26), loading the suspension onto a hemacytometer, and counting cells using a light microscope.

### Cytokine ELISA

Sandwich ELISAs measured IL-5, IL-13, and IFN-γ (27). ELISA plates were coated with anti-cytokine antibodies and incubated overnight at 4 °C. Plates were blocked for 30 min at room temperature with 150 μl of 20% FCS PBS containing 0.04% TWEEN 20 (FCS-PBST). Supernatants from lung cell restimulations were diluted in 5% FCS RPMI and added at 50 μl per well. After overnight incubation at 4 °C and washing, second-step antibody diluted in FCS-PBST was added at 50 μl per well. Plates were incubated for 1 h and washed, and then the enzyme conjugate diluted in FCS-PBST was added and incubated for another hour at room temperature. After washing, 100 μl of substrate (1 mg/ml 2,2′-azinobis(3-ethylbenzthiazolinesulfonic acid), 0.003% H_2_O_2_ in 0.1 M Na_2_HPO_4_, and 0.05 M citric acid) was added per well. The reaction was stopped by adding 0.05 ml of 0.2 M citric acid solution. The plates were read on an ELISA reader (Molecular Devices) at wavelengths Λ1-Λ2, where Λ1 equaled 405 and Λ2 was 490.

### Analysis of OVA-specific serum IgE

OVA-specific IgE was determined using a two-step sandwich ELISA (28). Plates were coated with 2 μg/ml of monoclonal anti-IgE antibody (EM95) overnight at 4 °C. Serum samples were added, followed by digoxigenin-coupled ovalbumin (DIG-OVA) from a kit containing digoxigenin-3-*O*-methylcarbonyl-ε−amonocaproic acid N-hydroxy-succinimide ester (Boehringer Mannheim Biochemica, Germany). Anti-digoxigenin-Fab fragments coupled to peroxidase (Boehringer) were added. After 1 h of incubation, 0.1 ml of substrate was added to each well.

### Analysis of KLH-specific serum IgE

KLH-specific IgE was determined using a two-step sandwich ELISA. Briefly, plates were coated with 2 μg/ml of EM95 overnight at 4 °C. A blocking solution made of 20% FCS in PBS containing polyoxyethylene sorbitan monolaurate (TWEEN 20) (Sigma, St Louis, MO) was added to each well for 1 h. After incubation, plates were washed with a solution of PBS-TWEEN 20 and serum samples and standards were added and incubated for 3 h at room temperature. Then, plates were washed and biotin-coupled KLH was added to each well. Plates were incubated for 1 h before washing, and horseradish peroxidase coupled to streptavidin was added. After 1 h of incubation, plates were washed again and 0.1 ml of substrate consisting of citric acid (Fisher Scientific, Fair Lawn, N.J) and sodium phosphate was added to each well. Plates were read on an ELISA reader.

### In-vitro restimulation of lung cells

Mice were euthanized, and lungs with lymph nodes were removed aseptically, cut into small pieces, and pressed through a sterile No. 100 steel mesh (Tylinter Inc., Mentor, OH) with a 10-ml plastic syringe. Cells were washed three times in Hanks’ balance salt solution (HBSS) solution and cultured in RPMI 1640 (J.H.R. Bioscience, Lenexa, KS) with 10% heat-inactivated FCS (Sigma), 0.05 mM 2-ME (Sigma), 2 mM L-glutamine (J.H.R. Bioscience), and penicillin/streptomycin (GIBCO BRL, Life Technologies Inc., Grand Island, NY). Lung cells were plated at 5 × 10^6^/ml in medium with 0.25 mg/ml OVA. Supernatants were harvested after 72 h for cytokine analysis.

### Measurement of OVA in the lungs of aerosolized mice

Immediately after a single OVA exposure, lungs were removed and a cell suspension prepared as above. The supernatant from the centrifugation was retained for quantification of OVA by an OVA-specific ELISA (23). Plates were coated with 5 μg/ml of rabbit anti-chicken egg albumin (Sigma Immunochemicals, St. Louis, MO.) in PBS and incubated overnight. After incubation, plates were blocked by adding FCS-PBST. Plates were washed, and samples were titrated on the test wells and incubated for a minimum of 3 h. Plates were washed, and 1 μg/ml of biotin-labeled rabbit anti-chicken egg albumin was added to each test well. After 1 h of incubation horseradish peroxidase-conjugated streptavidin was added to each well. After incubation for 1 h, substrate was added and plates developed as described for antibody ELISA.

### Analysis of IL-4

IL-4 from cultured supernatants was detected by bioassay using the IL-4-dependent CT.4S cell line (gift from Dr. William Paul, NIH). Cells were counted, washed, and resuspended in culture medium at 1 × 10^5^ per ml. Standards and test supernatants were titrated onto 96-well plates at 50 μl per well (range: 100 to 0.78 pg/ml). CT.4S cells were added at 50 μl per well, and plates were incubated for 72 h at 37 °C with 5% CO_2_. After incubation, 10 μl of 3-(4,5-dimethylthiazol-2-yl)-2,5-diphenyl tetrazolium bromide (Sigma) was added per well and incubated for 4 more hours at 37 °C. Then, 150 μl of 0.04N HCl in isopropanol was added, and absorbance measured at 570 and 650 nm.

### Sample size and statistics

The number of animals in each experimental group is reported in the corresponding figure legends. Sample sizes were determined based on prior studies utilizing similar aerosol sensitization protocols and the availability of age-matched animals. Thus, group sizes ranged n=4–10 mice per group depending on the experiment. OVA-specific IgE antibodies, eosinophils, and cytokine levels were calculated as mean and standard error of the mean (SEM). Two-tailed p values were determined using the Mann–Whitney Test; p < 0.05 was considered statistically significant.

## Results

### Aerosol-induced tolerance develops gradually with age

To study the development of aerosol OVA-induced tolerance, mice ranging from 3 days to 6 weeks of age were exposed to 20 min of 1% aerosolized OVA for 5 days beginning at day 0 as described in **Figure 1**. On day 45, all mice were challenged with an immunogenic dose of OVA. That is, when all neonatal mice reached adulthood, they were given an IP injection of 10 μg OVA/AL. This was followed by a single exposure to 1% aerosolized OVA for 20 min on day 60. Two groups of positive controls were used in this experiment, namely, the PBS controls and the OVA/AL controls. The PBS-positive controls were mice that were exposed to aerosolized PBS for 5 days beginning on day 0 followed by an injection of OVA/AL IP on day 45 and one dose of aerosolized OVA on day 60. The OVA/AL-positive controls were adult mice that were given an initial exposure of 10 μg OVA/AL IP on day 0 followed by a second dose on day 45 and aerosolized OVA on day 60. This group represented the maximal immunogenic stimulation in allergic mice.

**Figure 1:**
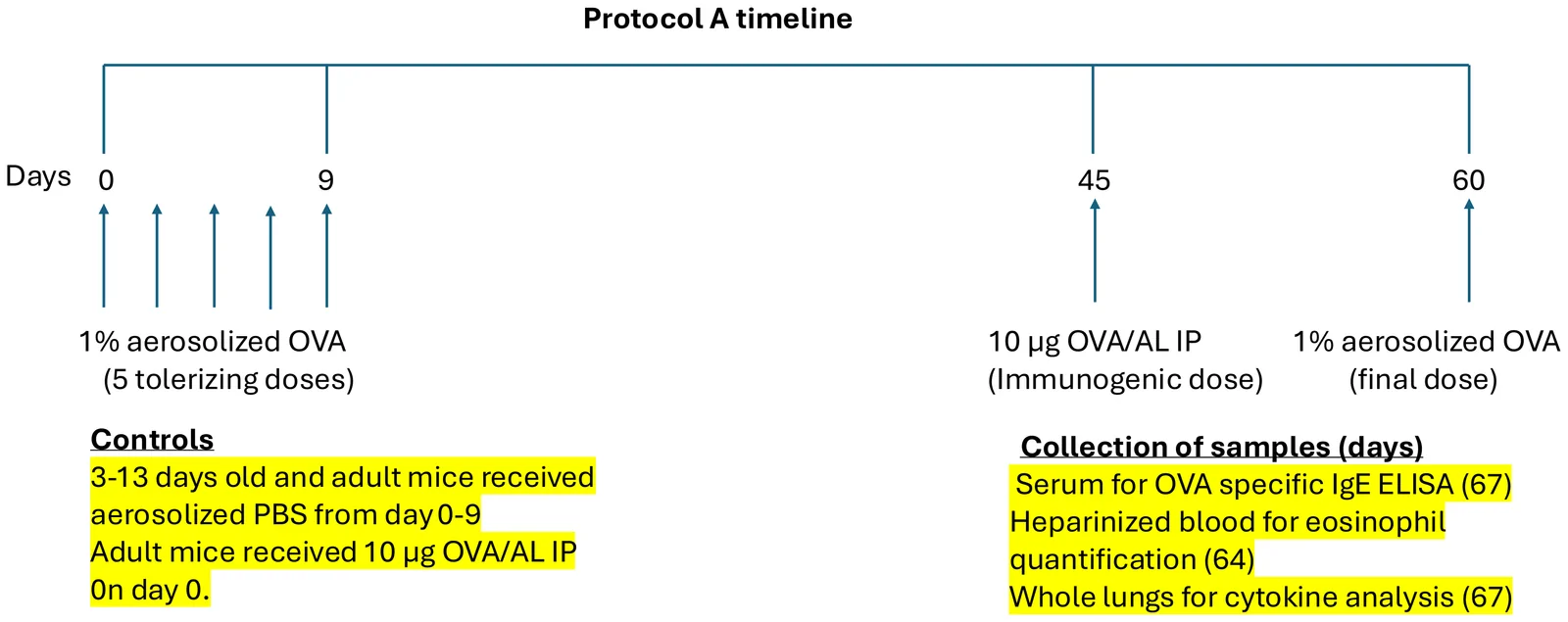
Antigen sensitization protocol of timeline A to evaluate the effect of age at initial aerosolized OVA exposure on subsequent allergic (immunogenic) challenges at adulthood.

Serum samples obtained on day 67 when all mice were at adulthood revealed elevated levels of OVA-specific IgE in mice initially exposed to aerosolized OVA at 3 days of age (**Figure 2**). There was a steady decline in these antibodies with the age of mice at the time of the first exposure to aerosolized OVA. That is, the older neonatal mice made less IgE antibodies at adulthood when compared with the younger neonatal mice. Specifically, the reduction of IgE was gradual with age at first exposure such that 13-day-old aerosol OVA-exposed weanlings (n=7) made a significantly (p<0.007) reduced level of OVA-specific IgE at adulthood when compared with adult mice (n=5) that were initially exposed to aerosol OVA at 3 days of age. The PBS-positive control group made significant levels of IgE, showing that one dose of OVA/AL IP was enough to prime mice for an allergic response. However, maximum OVA-specific IgE was seen in mice that were exposed to two doses of OVA/AL IP followed by 1% aerosolized OVA.

**Figure 2:**
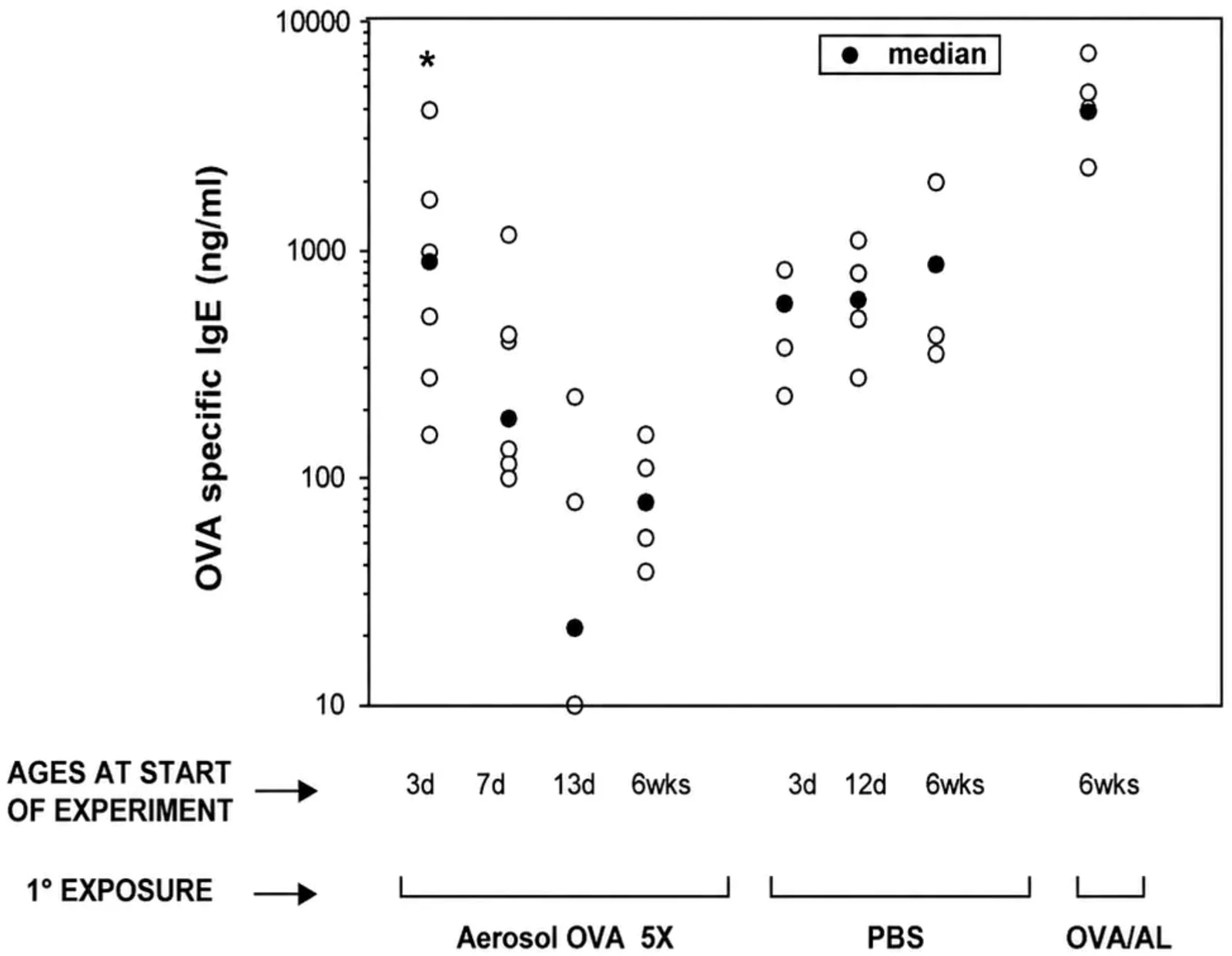
OVA specific IgE antibody production in BALB/c mice after exposure to OVA as described in **Figure 1**. Mice were bled from their tail veins on day 67. OVA-specific IgE antibodies were estimated by ELISA and the range of individual responses (open circles) and median (closed circles) are shown. * indicates p < 0.007 (n=7) vs the 13 days (n=7) and 6 weeks (n=5) old groups.

The levels of eosinophils in the blood of adult mice exposed to aerosolized OVA during the neonatal and the weanling periods were examined using the timeline A from **Figure 1**. Blood samples, taken on day 64, revealed an elevation of eosinophils in adult mice that were first exposed to aerosolized OVA at neonate (**Figure 3**). We had previously shown that adult naive mice exposed to aerosolized OVA made reduced levels of eosinophils when compared with naive adult mice that were given OVA/AL IP injection (23). However, in this study, it was found that adult mice exposed to aerosolized OVA as neonates (specifically 3 and 7 days old) made similar levels of eosinophils as the PBS-treated positive control group. Adult mice showed a gradual decline of eosinophil levels, which corresponded with the age of initial aerosol OVA exposure as observed in their OVA-specific IgE responses (**Figure 2**). Interestingly, the eosinophil concentration was significantly different when adult mice (n=5) were first exposed to aerosolized OVA compared with the adults that were first exposed as weanlings (18 days old; n=4) (p=0.006).

**Figure 3:**
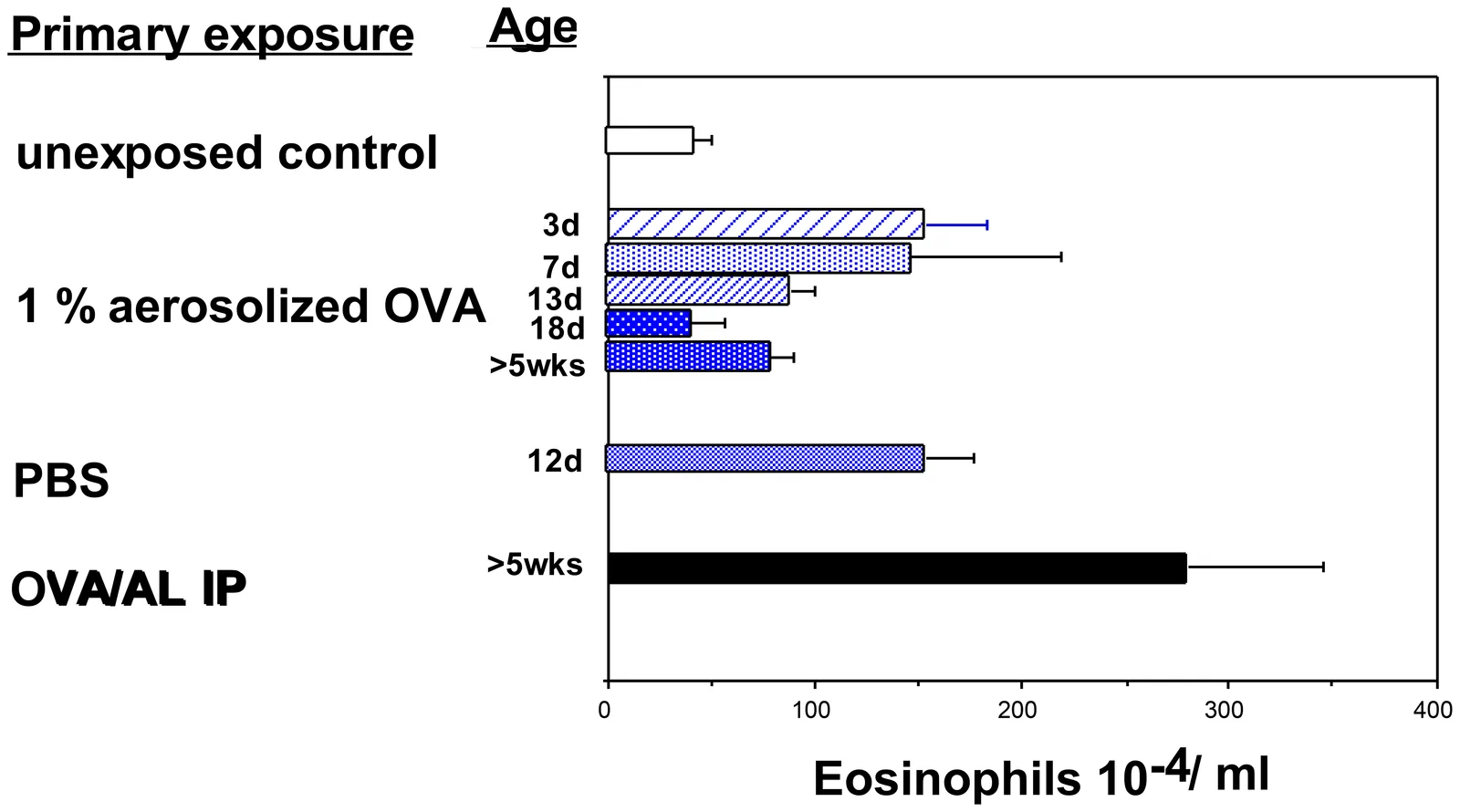
Blood eosinophils in OVA-sensitized mice. Experimental protocol was as shown in **Figure 1**. BALB/c mice were bled from the tail vein on day 64 and the eosinophils were counted in Discombe's fluid on a hemacytometer (26). Data represents the mean (± SEM) eosinophil counts per milliliter in mice. When the 18d group (n=4) was compared to the >5wks aerosol OVA primary exposure group (n=5), the probability value was p=0.006.

To determine whether Th2 responses were elevated in mice initially exposed to aerosolized OVA at 3 days old, cytokines in the lung were measured. Thus, lungs were removed and restimulated *in vitro* with OVA on day 67 after the completion of the timeline described in **Figure 1**. Pooled cells from the homogenized lung and associated lymph nodes were used for *in vitro* restimulations. It was observed that the positive control mice primed by OVA/AL IP or PBS made elevated levels of IL-4, IL-5, and IL-13 and undetectable levels of IFN-γ (**Table 1**). There was no substantial elevation in Th2 cytokines in adult mice initially exposed to aerosolized OVA as weanlings (13 days old) or adults. However, adult mice (>5 weeks old) initially exposed to aerosolized OVA at 3 and 7 days of age made substantial Th2 cytokine responses. The responses in the mice initially exposed to aerosolized OVA at 3 days of age were comparable with the PBS-positive control group. Adult mice initially exposed to aerosolized OVA at 7 days of age made Th2 cytokines, but their cytokine responses were reduced when compared with mice initially exposed to aerosolized OVA at 3 days of age. Except for IL13, cytokines were not detected in the lungs of adult mice that were first exposed to aerosolized OVA at 18 days of age. However, there were very low but detectable levels of Th2 cytokines in the lung of mice that were first exposed to OVA by aerosolization as adults.

**Table 1:** OVA-specific lung cytokines in mice exposed to aerosolized OVA.

Ages of mice at primary exposure	Primary exposure	IL13 (pg/ml)	IL4 (pg/ml)	IL5 (ng/ml)	IFN-γ (ng/ml)
3 days	1% aerosolized OVA	1958 ±333	66±12	0.9±0.1	<0.16
7 days	1% aerosolized OVA	912±282	25±4	0.8±0.1	<0.16
13 days	1% aerosolized OVA	<1.95	<16	<0.156	<0.16
18 days	1% aerosolized OVA	14±8	<16	<0.156	<0.16
6 weeks	1% aerosolized OVA	148±27	<16	0.3±0.06	<0.16
3 days	PBS	784±359	37±11	0.4±0.18	<0.16
6 weeks	PBS	1929±327	34±2	0.9±0.1	<0.16
6 weeks	10 ug OVA/AL IP day 0	3692±236	105±11	2.8±0.3	<0.16

Mice ranging in age from 3 days to 6 weeks of age were exposed to 5 doses of aerosolized OVA as shown in **Figure 1**, timeline A while 2 control groups received aerosolized PBS. The positive control group received 10ug OVA/AL IP on day 0. On day 45, all mice received an immunogenic dose of 10ug OVA/AL IP followed by a 1% aerosolized OVA on day 60. Lungs were harvested on day 67, homogenized and restimulated in vitro using whole lung cells at a concentration of 5 x 10^6^ cells per ml with 0.25 mg/ml OVA in culture medium. Supernatants were harvested after 72 hours and tested for cytokines.

### Aerosol OVA exposure during the neonatal period prevented mice from being tolerized by a second series of aerosol OVA exposures that began at 6 weeks of age

Having shown that 3-day-old naïve mice exposed to aerosol OVA make an allergic response after immunogenic doses of OVA at adulthood, the ability for 3-day-old aerosol OVA-exposed mice to become tolerized upon further exposure to aerosol OVA at adulthood (6 weeks of age) was examined. Thus, as shown in **Figure 4**, mice ranging in age from 3 days to 6 weeks old were exposed to five doses of 1% aerosolized OVA from day 0 through day 9. At adulthood (day 45-day 53), they were reexposed to five more doses of a 1% aerosolized OVA. This was followed by immunogenic doses of OVA/AL IP on day 67 and a single dose of aerosol OVA on day 81. The PBS-positive controls were 6-week-old mice that received five doses of aerosol PBS from days 0 to 9 and again from days 45 to 53. This group received the same doses of OVA as given to the experimental mice on days 67 and 81. The OVA/AL IP-positive control group were adults at the start of the experiment. They received 10 μg OVA/AL IP on days 0 and 67 followed by a single dose of aerosolized OVA on day 81.

**Figure 4:**
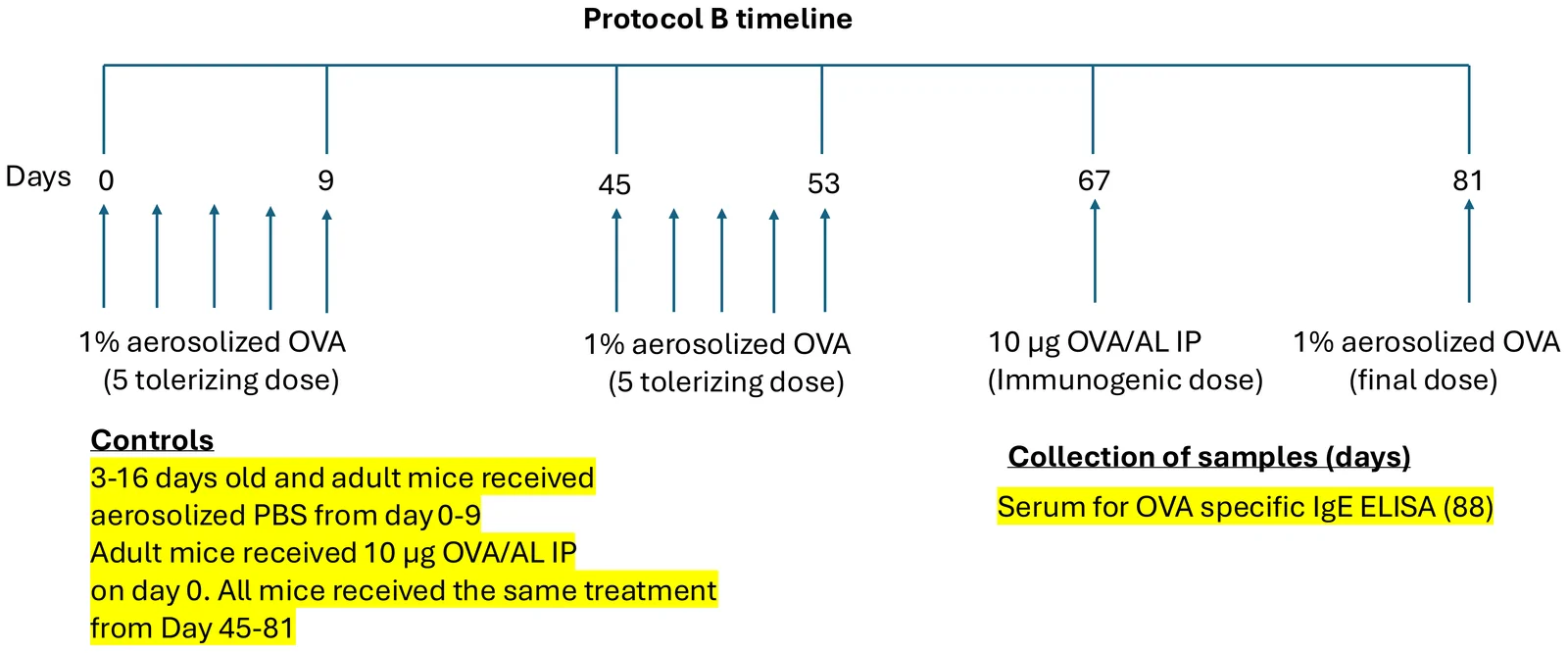
Timeline of protocol B examined whether neonatal exposure altered the ability of mice to develop inhalation tolerance following repeated aerosolized OVA exposures in adulthood.

The results in **Figure 5** show the OVA-specific IgE levels in mice on day 88 (7 days after the last aerosol OVA). Experimental mice were unresponsive with respect to OVA-specific IgE even after immunogenic challenges of the antigen except when the mice were exposed to aerosolized OVA at 3 days of age. There were no detectable OVA-specific IgE antibodies in aerosolized OVA mice initially exposed to the antigen older than 1 week of age.

**Figure 5:**
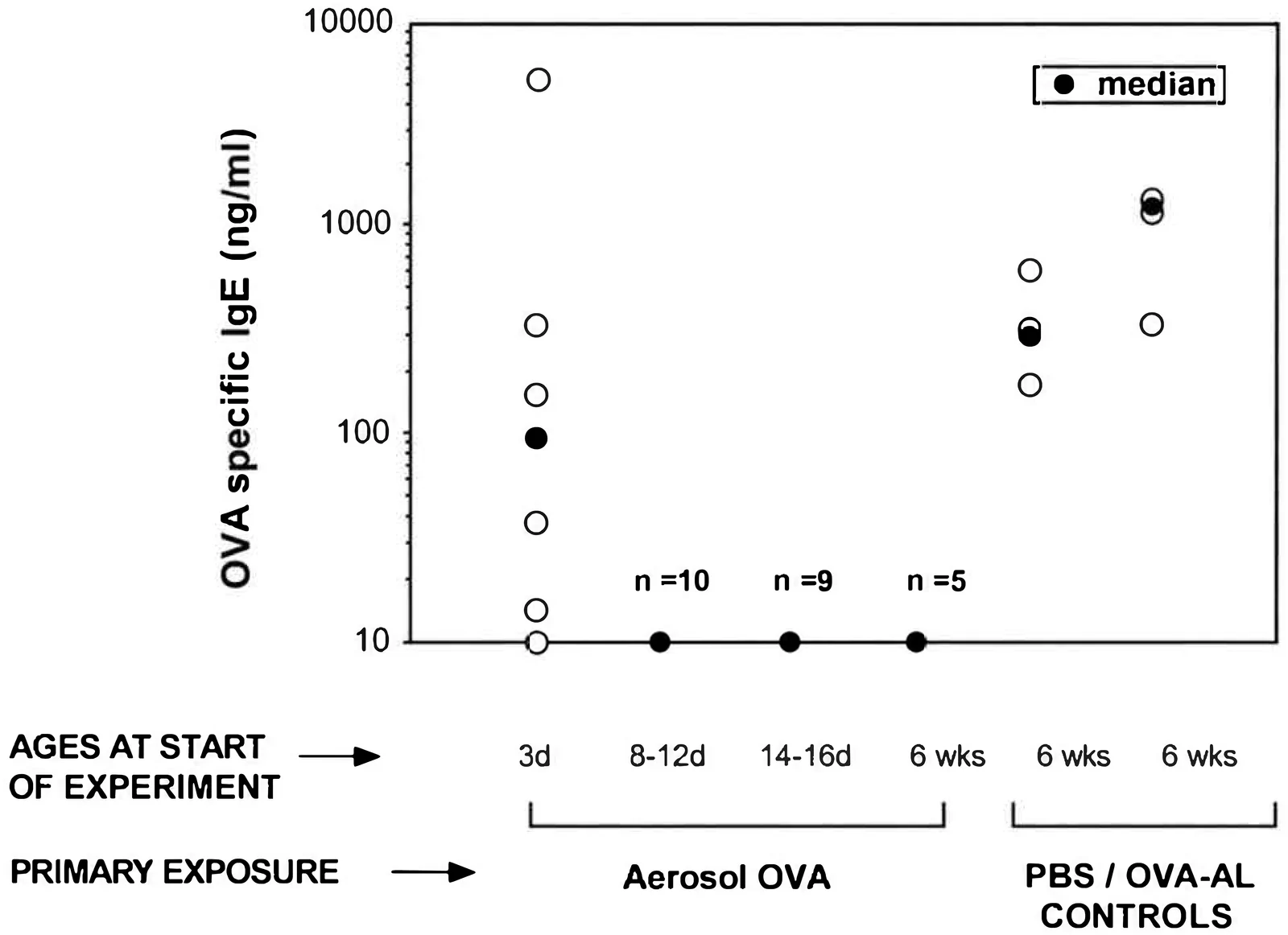
IgE anti-OVA specific antibodies in adult mice exposed to aerosolized OVA at neonate followed by tolerizing doses at adulthood as shown in **Figure 7**. Mice were bled from their tail veins on day 88. OVA-specific antibodies were estimated by ELISA and the range of individual responses (open circles) and median (closed circles) are shown.

The amounts of OVA that is delivered into the lung of mice after 20 min to 1% aerosolized OVA were examined (**Figure 6**). Here, it was observed that adult mice received the highest amount of OVA (1,731 ± 290 ng) with a steady decline in intake with age. Initially, a 2-day-old mouse received 232 ± 69 ng at the start of the aerosol OVA exposure and 396 ± 70 ng at 10 days of age. Therefore, five exposures of aerosolized OVA when begun in 2-day-old mice received approximately the same amount of OVA as a single aerosol exposure given to a weanling or adult mouse.

**Figure 6:**
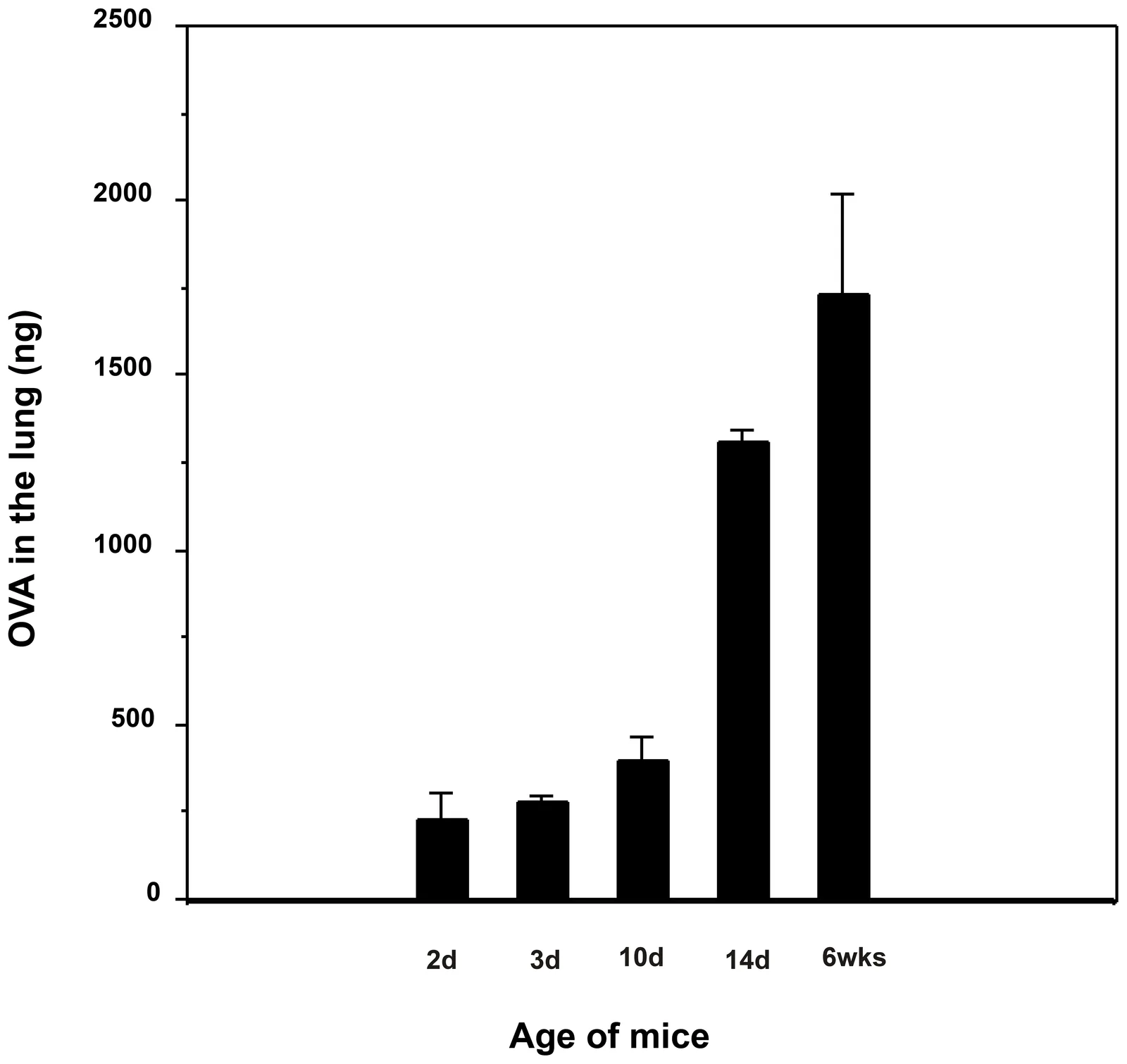
IgE anti-OVA specific antibodies in adult mice exposed to aerosolized OVA at neonate followed by tolerizing doses at adulthood as described in timeline B of **Figure 4**. Mice were bled from their tail veins on day 88. OVA-specific antibodies were estimated by ELISA and the range of individual responses (open circles) and median (closed circles) are shown.

### A single exposure of aerosolized OVA to 2-day-old mice was enough to prevent the establishment of complete tolerance at adulthood

We have previously shown that IgE unresponsiveness can be induced in adult mice after they were exposed to small doses (0.01%) of aerosolized OVA, which is equivalent to approximately 34 ng (23). In this study, the effects of a single exposure of 1% of aerosol OVA for 20 min during the neonatal period were investigated. The experimental group received a single exposure to 1% aerosolized OVA for 20 min at 2 days of age followed by the tolerizing doses from days 45 to 53, as shown in timeline C of **Figure 7**. This was followed by immunogenic doses of 10 μg OVA/AL IP on day 67 and a single challenge of aerosolized OVA at day 81. The group that was made unresponsive to IgE was exposed to PBS on day 0, but the other exposures (days 45-81) were the same as the experimental group. The positive control group was exposed to PBS on day 0 and again from days 45 to 53. This group received 10 μg OVA/AL IP on day 67 and a single challenge of aerosolized OVA at day 81.

**Figure 7:**
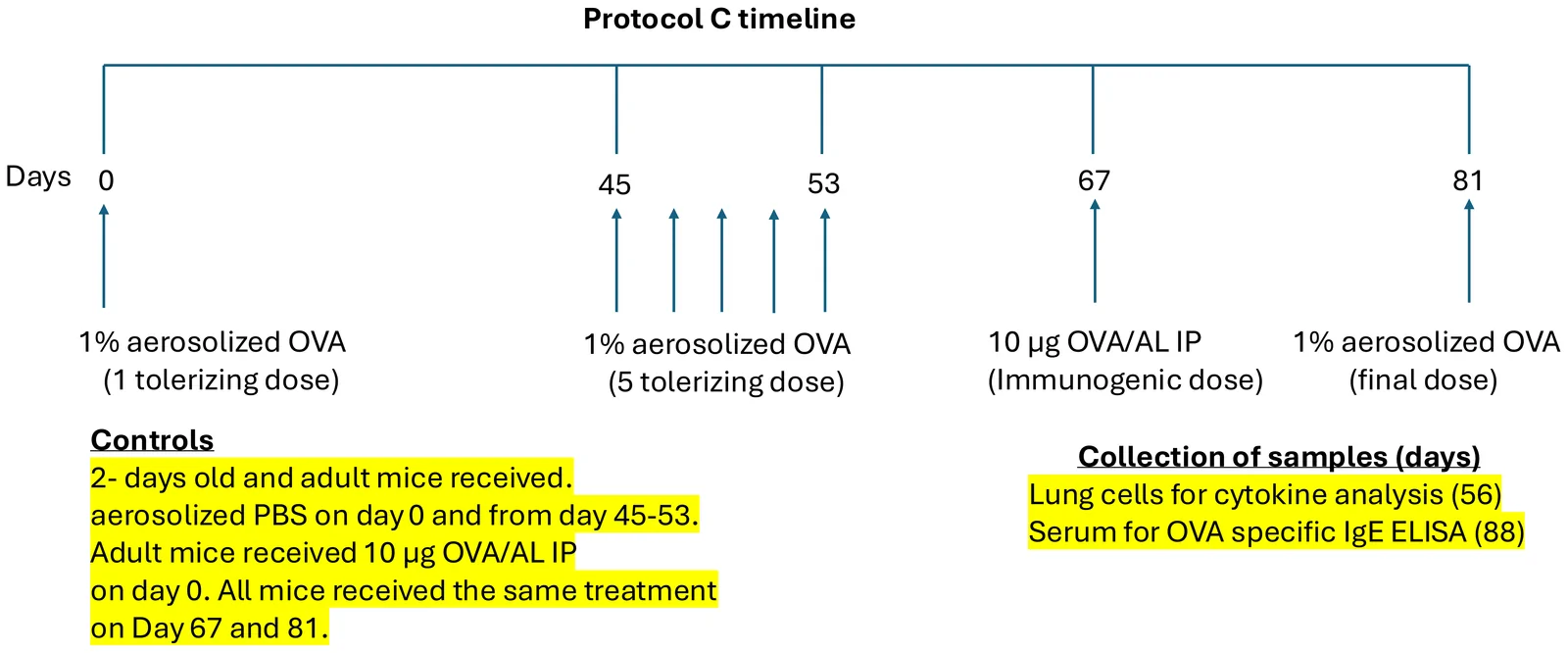
Timeline C of the experimental protocol which investigated whether a single neonatal aerosol exposure was sufficient to alter tolerance development and whether blockade of IL-4 signaling during neonatal exposure modified these outcomes.

A single exposure of aerosolized OVA to neonatal mice (specifically 2-day-old mice) prevented the establishment of complete tolerance at adulthood. The results of nine experiments were summarized, and a wide variation of the IgE responsiveness in mice initially exposed to the antigen at 2 days of age were observed (**Figure 8**). 35% of the mice initially exposed to aerosolized OVA at 2 days of age had low or undetectable levels of OVA-specific IgE, whereas 65% made greater than 100 ng/ml of OVA-specific IgE antibodies. 74% of the mice from the tolerized group made less than 20 ng/ml OVA-specific IgE, and the remaining mice made low levels of OVA-specific IgE which was between the range of 20 and 99 ng/ml. It was also observed that neonatal mice (2 days old) exposed to aerosolized OVA followed by repeated doses to aerosolized OVA when begun 6 months later were also prevented from being IgE unresponsive (data not shown).

**Figure 8:**
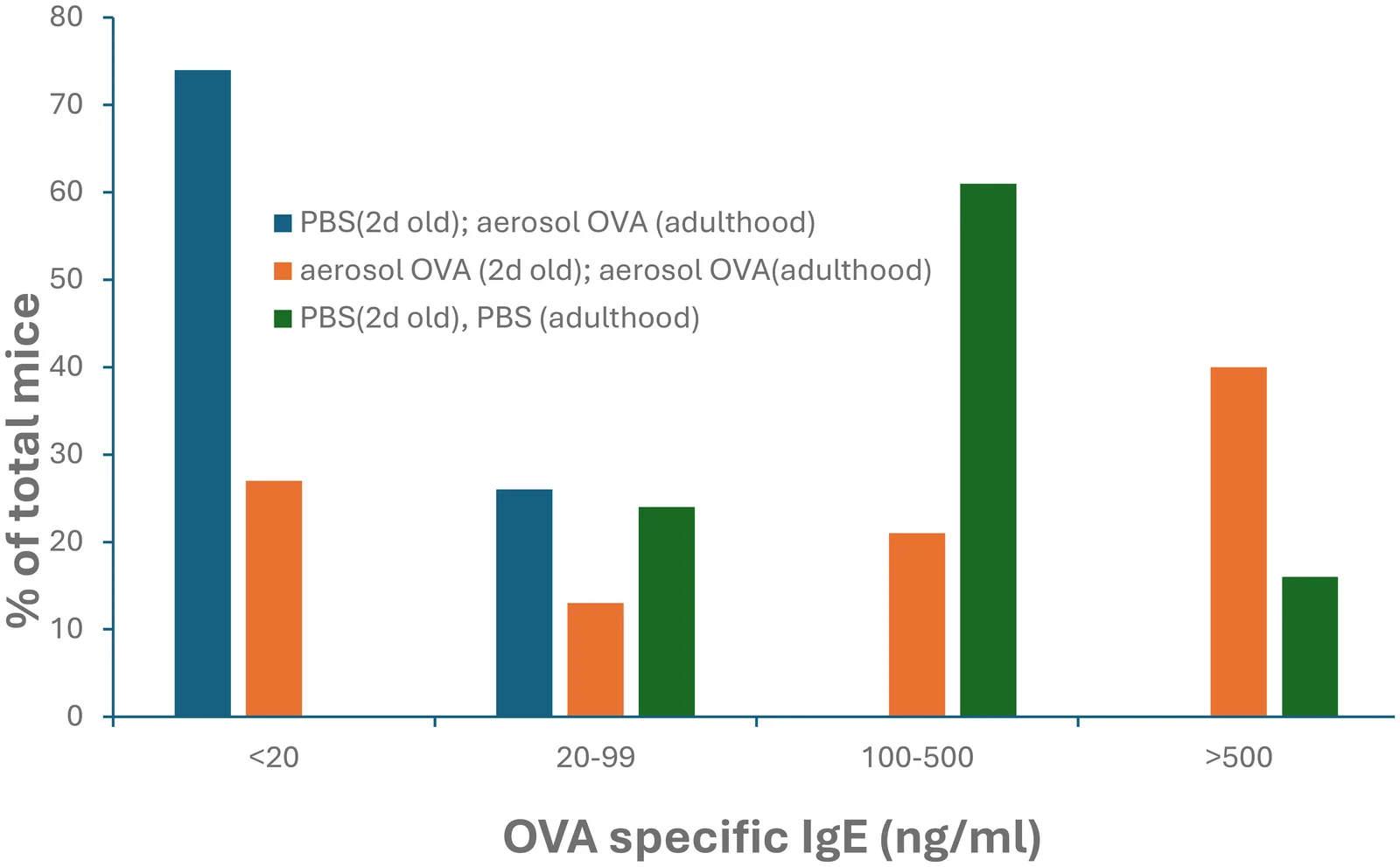
A single aerosol exposure to OVA at day 0 primes for a strong Th2 response. The experimental protocol was as described in protocol C of **Figure 7**. Mice were bled on day 88, and anti-IgE OVA-specific antibodies were determined. OVA-specific antibodies were estimated by ELISA. Control mice were exposed to aerosolized PBS on day 0, and days 45–53 or were given 10 μg OVA/AL on day 45. All controls also received 10 μg OVA/AL IP on day 67 and 1% aerosolized OVA for 20 min on day 81. The histogram shows the range of the OVA-specific IgE antibodies on day 88 from a pool of nine experiments.

### Th2 cytokines were observed in the lung of mice exposed to a single dose of aerosolized OVA at neonate followed by tolerizing doses of OVA at adulthood

To examine the effect of the early exposure of OVA in neonatal mice, the effect of the tolerizing doses of aerosolized OVA in mice previously exposed to a single dose of aerosolized OVA was examined. Thus, 2-day-old and adult mice were exposed to a single exposure of aerosolized OVA or PBS on day 0. At adulthood (days 45-53), all mice received five doses of aerosolized OVA (**Figure 7**). On day 56, lungs were removed, homogenized, and restimulated *in vitro* with OVA. Pooled cells from the lungs and associated lymph nodes were used for the *in vitro* restimulation. There were no detectable OVA-specific IgE in the serum of mice; however, elevated levels of Th2 cytokines (IL4, IL5, and IL13) in the lung supernatant of mice initially exposed to aerosolized OVA at 2 days of age were observed (**Table 2**). The mice initially exposed to aerosolized OVA as adults were unresponsive with respect to cytokines tested. Thus, the administration of aerosolized OVA at neonate (2 days old) of age primed mice for a Th2 response upon subsequent exposure to aerosolized OVA.

**Table 2:** Cytokines in the lungs of adult mice exposed to aerosolized OVA during the neonatal period.

Primary exposure on day 0 (age)	Secondary exposure from d45–53 (at adulthood)	IL13 (pg/ml)	IL4 (pg/ml)	IL5 (pg/ml)	IFN-γ (pg/ml)
1%aerosolized OVA (2 days old)	1% aerosolized OVA	1736 ±416	39±14	1329±290	<78
PBS (2 days old)	1% aerosolized OVA	<2.0	<1.6	<78	<78
1% aerosolized OVA (adults)	1% aerosolized OVA	<2.0	<1.6	<78	<78

Mice were exposed to a single dose of aerosolized OVA on day 0 while a control group received aerosolized PBS. All mice received aerosolized OVA for 5 days from day 45-53. On day 56 lungs were removed, homogenized and in vitro restimulation of lung cells were carried out at a concentration of 5 x 10^6^ cells per ml with 0.25 mg/ml OVA. Supernatants were harvested after 72 hours.

### Administration of anti-IL4 to neonates exposed to aerosolized OVA restores the dysregulated immune response at adulthood

Since IL-4 is known to regulate Th2 responses, we examined the presence of the early production of IL-4 during exposure to aerosolized OVA. Here, neonatal mice were exposed to aerosolized OVA and were given 0.5 mg of anti-IL4 or GL113, the control antibody, at the time of the exposure. The protocol is as described in **Figure 7** but also included the administration of antibodies on day 0. The goal was to examine the role of the early production of IL4 in priming mice for an allergic response at adulthood. It was observed that 2-day-old mice treated with anti-IL4 made significantly reduced levels of OVA-specific IgE at adulthood when compared with those treated with the control antibody (**Figure 9**). That is, the control antibody treated-aerosolized OVA exposed group made OVA-specific IgE at adulthood with a median (368 ng/ml) that was similar to the PBS-positive control groups primed with OVA/AL IP. However, the aerosolized OVA 2-day-old group treated with anti-IL4 made reduced levels of OVA-specific IgE at adulthood. Here, the median response was less than 10 ng/ml (p=0.015, n=6 when compared with the group that was administered GL113 and aerosolized OVA at 2 days of age, n=5). This result shows that administration of anti-IL4 to 2-day-old mice exposed to aerosolized OVA caused them to be IgE unresponsive when exposed to tolerizing doses of OVA at adulthood.

**Figure 9:**
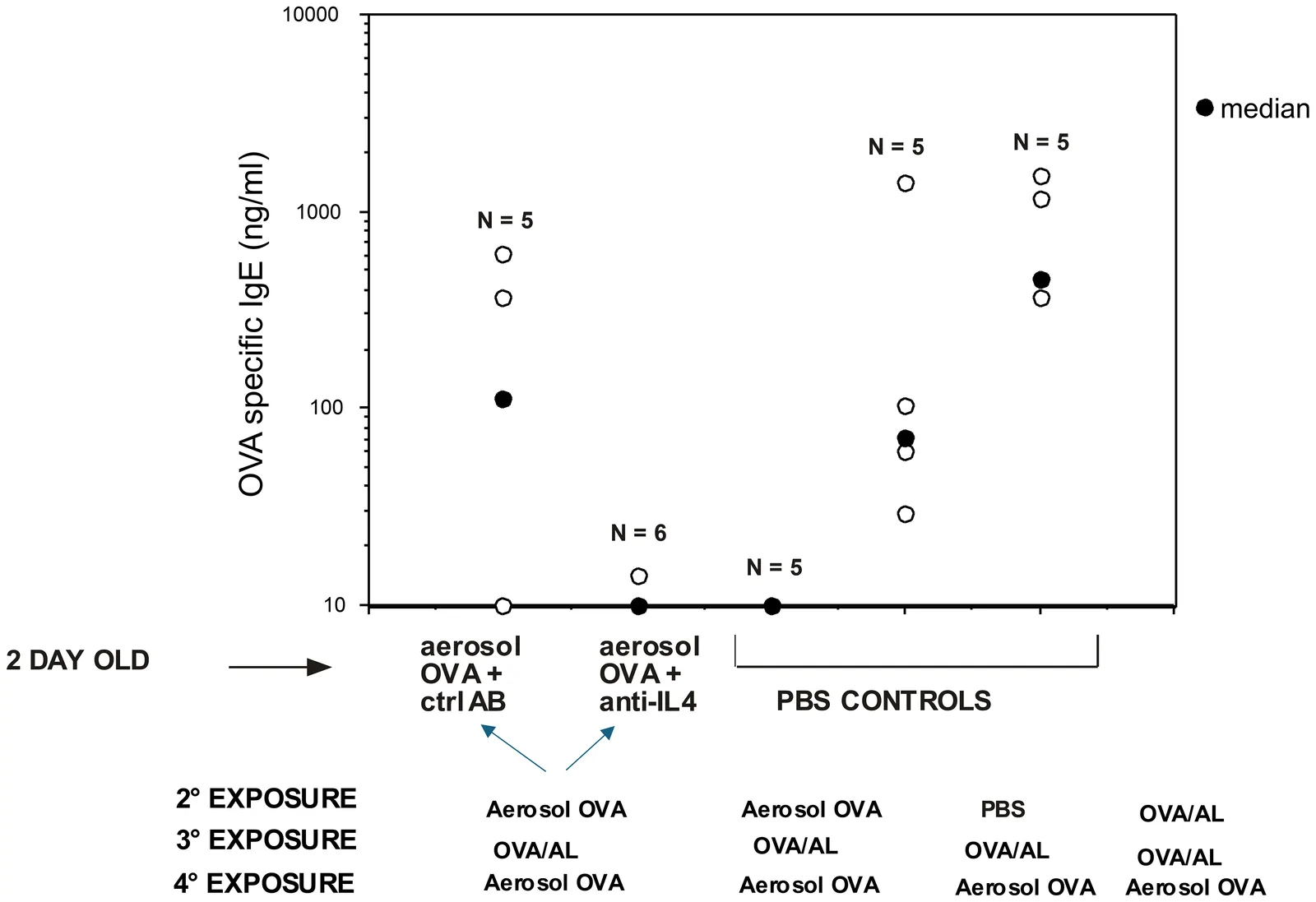
The administration of anti-IL4 to 2-day-old mice at the time of aerosol OVA exposure induces IgE unresponsiveness after tolerizing doses of aerosolized OVA at adulthood. Naïve 2-day-old BALB/c mice were given either 0.5 mg of anti-IL4 IP and exposed to 1% aerosolized OVA or PBS for 20 min. The control was 2-day-old mice injected with a control antibody IP (GL113) and exposed to aerosolized OVA for 20 min. At 6 weeks of age, they were exposed to five doses of 1% aerosolized OVA every other day for 10 days. This was followed by a single dose of OVA/AL IP and 1% aerosolized OVA as shown in **Figure 7**. p = 0.015 when the anti-IL4-treated group was compared with the GL113 group.

The ability of adult mice to mount a strong immunogenic response to OVA after the depletion of IL4 during the neonatal period was examined. Thus, 2-day-old mice were exposed to a single dose of a 1% aerosolized OVA for 20 min and treated with either anti-IL4 or GL113. At 6 and 8 weeks old, they were given immunogenic doses of OVA/AL IP followed by an exposure to aerosolized OVA (**Figure 10**). Here, it was observed that mice that were exposed to aerosolized OVA at 2 days of age made significantly more OVA-specific IgE at adulthood when compared with those exposed to PBS as neonates but primed as adults (p < 0.04, n = 5). However, if aerosol OVA-exposed neonatal mice were treated with anti-IL4 and given the same strong immunogenic doses of OVA/AL IP, they made OVA-specific IgE which was similar to the PBS-positive control group. The dysregulated OVA-specific IgE response from mice exposed to aerosol OVA at neonate was prevented by the injection of anti-IL4 at the time of the initial OVA exposure. These results show that the depletion of IL4 in neonatal mice does not impair their ability to mount a normal OVA-specific IgE immune response at adulthood.

**Figure 10:**
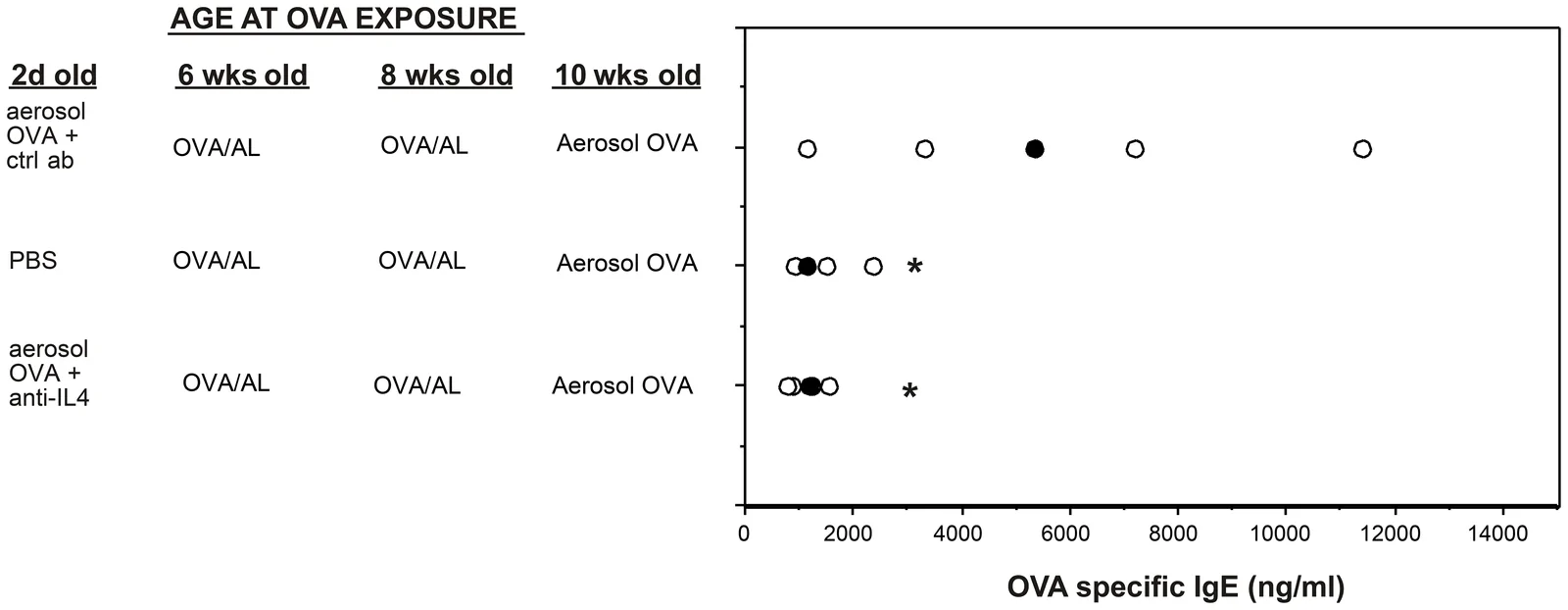
The exposure of 2-day old mice to aerosolized OVA significantly enhances the Th2 response upon exposure to immunogenic doses of OVA in adulthood. Naïve 2-day old BALB/c mice were given either 0.5 mg of anti-IL4 IP or GL113 and exposed to a 1% aerosolized OVA for 20 mins. At 6 and 8 weeks of age they were injected with 10 µg of OVA/AL. This was followed by a single exposure of a 1% aerosolized OVA for 20 mins at 10 weeks of age. *indicates that p < 0.04 vs the aerosol OVA control antibody group. n = 5 for all groups.

Finally, we addressed the issue of the overall immune status of adult mice after treatment with anti-IL4 at the neonatal stage. Mice that were given anti-IL4 on day 0 were exposed to OVA as described in the protocol of **Figure 7**. However, on days 133 and 140, all mice were given an IP injection of the immunogen TNP-KLH. Seven days after the last injection of TNP-KLH, mice were bled and tested for their ability to mount a KLH-specific IgE response. We found that there were no significant differences in the KLH-specific IgE antibodies from the mice that were treated with anti-IL4 at neonate when compared with the untreated groups (**Figure 11**).

**Figure 11:**
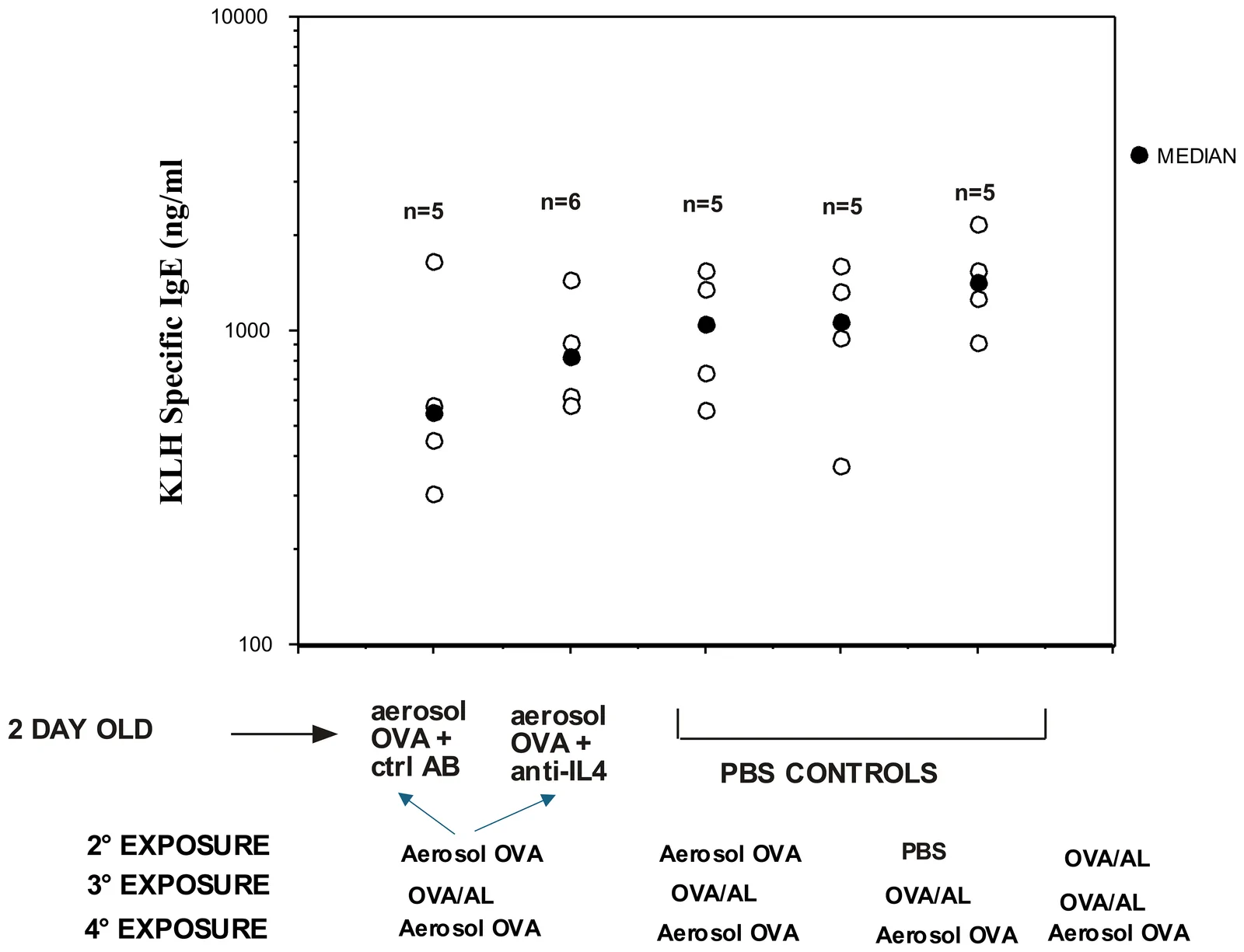
Neonatal mice treated with anti-IL4 and exposed to aerosolized OVA made a normal immune response to allergenic doses of an irrelevant antigen such as TNP-KLH. Mice that were given anti-IL4 on day 0 were exposed to OVA as described in the protocol of **Figure 7C**. On days 133 and 140, all mice were given an IP injection of the antigen TNP-KLH. Seven days after the last injection of TNP-KLH, mice were bled and tested by ELISA for their ability to mount a KLH-specific IgE response.

## Discussion

In this report, the development of aerosol-induced IgE tolerance was investigated. Our previous study had shown that naïve adult mice exposed to aerosolized OVA became tolerant with respect to OVA-specific IgE despite further administration of immunogenic doses of OVA (23). It can be accomplished in the absence of IFN-γ, and it does not require CD8 or γδ cells. Additionally, we found that OVA-specific IgE tolerance persists in IL10−/− (deficient) mice as well as in mice lacking IL12 or nitric oxide (BWP Seymour unpublished data). However, the results of many epidemiological investigations have implied that allergies and allergic asthma occur in early life (29–35). These observations led us to question the timing of the development of the inhalation tolerance mechanism in a murine model of allergic sensitization.

The first series of experiments in this study were done to determine the age of mice at the onset of the development of this inhalation tolerance mechanism. Here, it was demonstrated that the age of mice at the time of the initial exposure to aerosolized OVA was a defining factor in determining the outcome of the response after immunogenic challenges at adulthood. That is, neonatal mice and weanlings (2–7 days old) exposed to aerosolized OVA will become allergic during adulthood if exposed to immunogenic doses of the same antigen later in life. These data confirm findings from several epidemiological studies suggesting that exposure to allergens during infancy and early childhood is a risk factor for allergic sensitization (31–36).

The principal finding of this work is that aerosol-induced tolerance develops in an age-dependent manner, with neonatal mice exhibiting a marked impairment in establishing tolerance following antigen exposure. Mice exposed to aerosolized OVA at 2–3 days of age developed robust OVA-specific IgE responses and eosinophilia at adulthood, whereas mice first exposed at later developmental stages demonstrated reduced IgE and eosinophil responses, consistent with progressive maturation of tolerogenic mechanisms. That is, the inhalation tolerance mechanism develops gradually with age.

It was also shown that during the weanling period, there is a partial reduction of these parameters in adults initially exposed to OVA. That is, this inhalation tolerance mechanism is non-existent in newborn, but it is partially developed by day 7 and is fully developed in 2-week-old weanlings. This suggests that the inhalation tolerance mechanism is delayed at birth, creating a window for increased risk for sensitization during the neonatal period. These data support the concept of a critical developmental window during the neonatal period when the immune system is particularly susceptible to allergic sensitization (37, 38).

The data demonstrate that aerosolized antigen exposure induces a state of immunologic tolerance in both adult and 2-week-old weanling mice; however, the degree and quality of this tolerance differ substantially by age at first exposure. Adult mice exposed to aerosolized OVA exhibit reduced Th2 responses, as evidenced by low IgE production and attenuated cytokine levels, even following systemic sensitization with OVA/AL IP, indicating the establishment of functional tolerance. Nevertheless, these mice retain low but detectable cytokine responses, suggesting that tolerance in adulthood is incomplete and compatible with residual immune activation. In contrast, mice first exposed during the weaning period (13–18 days of age) demonstrate a markedly deeper level of suppression, characterized by near-complete absence of Th2 cytokines (IL-4, IL-5, and IL-13), lack of IL-10 upregulation (data not shown), and minimal IgE and eosinophilic responses. This pattern indicates that tolerance induced during the weaning window is not simply mediated by classical cytokine-driven regulation but rather reflects a more profound state of antigen-specific unresponsiveness. Mechanistically, this may involve deletion or long-term functional inactivation of antigen-specific T cells, reduced costimulatory capacity of antigen-presenting cells, and enhanced tolerogenic programming associated with immune maturation and microbiome expansion during this developmental stage. Consequently, antigen exposure during the weaning period results in a more complete and durable suppression of allergic sensitization than exposure in adulthood, where tolerance, although present, remains partial.

An important observation from this study is that a single neonatal exposure to aerosolized antigen is sufficient to disrupt tolerance induction, even when subsequent exposures are delivered under tolerizing conditions. Exposure of neonatal mice to a single dose of aerosolized OVA of less than 200 ng of OVA was sufficient to prevent aerosol-induced tolerance and ultimately led to priming for a Th2 response. This study reveals substantial heterogeneity in IgE responsiveness among neonatally exposed mice, with a subset developing high IgE levels, whereas others remained low responders. This variability suggests that additional factors—potentially including stochastic immune events, microenvironmental influences, or early innate immune activation—may influence the trajectory of allergic sensitization. Nonetheless, the overall shift toward increased IgE production in neonatally exposed mice underscores the dominant effect of early antigenic exposure on immune programming.

Il-4 signaling during neonatal exposure is required for the establishment of a dysregulated allergic phenotype in adulthood. It is a key regulator of Th2 differentiation through STAT6 activation and GATA3 induction, and its role in allergic inflammation is well established (13, 23, 37, 39–41). This study identifies neonatal IL-4 signaling as an important contributor to the development of long-term allergic responses following early-life antigen exposure. The finding that transient IL-4 blockade during neonatal exposure reduced subsequent allergic sensitization supports a role in shaping later immune outcomes. However, additional studies will be required to define the precise cellular and molecular mechanisms through which early IL-4 signaling influences long-term susceptibility to allergic diseases. Critically, the blockade of IL-4 at the time of neonatal antigen exposure restored the ability of mice to develop tolerance, as evidenced by significantly reduced OVA-specific IgE levels at adulthood. Notably, anti-IL-4 treatment did not impair the capacity of mice to mount antigen-specific IgE responses following strong immunogenic challenge, indicating that IL-4 depletion selectively prevents maladaptive priming without inducing global immunosuppression. That is, the exposure to OVA at neonate had no effect on the development of the inhalation tolerance mechanism due to the removal of IL4. These results establish early IL-4 signaling as a non-redundant and temporally restricted driver of allergic sensitization and suggest that modulation of this pathway during early life may represent a viable strategy for preventing allergic disease in atopic individuals. This early presence of IL-4 is therefore a plausible explanation for epidemiologic studies that show that events occurring early in life are significantly associated with the risk of asthma and poor lung function later in life (30, 33, 34).

In addition to IL-4, elevated IL-5 production was associated with persistent eosinophilic inflammation in mice exposed to allergen during the neonatal period. These data support a role for IL-5 in sustaining eosinophilic inflammation. Given the established role of IL-5 in eosinophil maturation, survival, and recruitment, these observations were consistent with a contribution of IL-5 to the observed phenotype. However, direct functional manipulation of IL-5 was not performed in the present study, and therefore causal conclusions regarding IL-5 signaling cannot be made. The persistence of eosinophilia further supports the concept that early immune events can induce durable changes in immune homeostasis, potentially through epigenetic modifications or long-lived memory responses (39). This finding contrasts with prior observations in adult models, where repeated low-dose exposure promotes tolerance (23–25). This indicates that the neonatal immune system is uniquely biased toward sensitization. Furthermore, neonatal exposure prevented tolerance even after delayed reexposure months later, suggesting that neonatal antigen exposure may have persistent effects on subsequent immune responses. While these findings are consistent with long-term immune programming, alternative explanations including prolonged allergen-specific memory responses or age-dependent differences in tolerance induction cannot be excluded.

Interestingly, analysis of cytokine responses revealed that mice exposed to inhaled antigens during the neonatal period exhibited robust Th2 cytokine production despite the absence of detectable serum IgE in some settings, indicating that cellular immune priming may precede or occur independently of humoral responses. This dissociation highlights the importance of evaluating both cellular and humoral compartments when assessing allergic sensitization and suggests that cytokine profiling may serve as a more sensitive indicator of early immune programming.

These findings suggest that early IL-4 production may contribute to enhanced allergic sensitization following neonatal allergen exposure. However, allergic disease is multifactorial, and the present data do not exclude contributions from additional cytokine pathways, environmental influences, genetic susceptibility, or developmental immune factors.

This early exposure to a specific aerosol antigen will lead to an augmented Th2 response in adulthood. This was demonstrated when mice exposed to aerosol PBS was compared with those exposed to aerosolized OVA at neonate followed by the maximal immunogenic doses at adulthood. Significant dysregulation was observed from the group exposed to aerosolized OVA as neonate. However, a single dose of anti-IL-4 at neonate during the time of the initial exposure to aerosolized OVA was necessary to induce a normal and not dysregulated Th2 response at adulthood. That is, this response was not dysregulated but was characteristic of an allergic response seen when naïve mice were made allergic by priming with OVA/AL IP.

A limitation of this study is the absence of a dedicated low-IgE negative control group in the experimental design shown in **Figure 10**. An appropriate negative control would have consisted of naïve adult mice initially exposed to aerosolized OVA at 6 weeks of age, followed by the same immunogenic OVA/AL sensitization protocol and OVA aerosol challenge performed at 8 and 10 weeks of age. Based on our previous studies using this experimental system, these animals would be expected to develop inhalational tolerance rather than allergic sensitization, with OVA-specific IgE concentrations typically remaining below 20 ng/mL. Inclusion of this group would have provided a direct reference for the low-IgE tolerant phenotype and further strengthened interpretation of the marked increase in OVA-specific IgE observed in neonatal mice receiving the control antibody. Nevertheless, the principal comparison in **Figure 10** remains valid, as neonatal IL-4 blockade restored antigen-specific IgE responses from the dysregulated, markedly elevated levels observed in control antibody-treated mice to levels characteristic of a normal immune response following conventional OVA/AL sensitization. Thus, while inclusion of the adult tolerant control would have strengthened the study, its absence does not alter the conclusion that transient neonatal IL-4 blockade prevents the development of the exaggerated allergic phenotype induced by neonatal aerosol exposure.

These findings have significant implications for understanding the origins of allergic disease in humans. The neonatal period represents a time of heightened immune plasticity, during which environmental exposures, including aeroallergens—may have disproportionate effects on long-term immune function. This study supports the hypothesis that early-life Th2-biased immune responses contribute to the development of atopy and asthma (36, 37). This work reinforces the concept that allergic disease originates during a period of heightened immune plasticity, during which targeted immunomodulation may alter the natural history of atopy and asthma (34, 39, 42). IL-4 emerges as a central upstream mediator of Th2 polarization, whereas downstream effector pathways—including IL-5–driven eosinophilic inflammation—represent key drivers of disease severity and persistence. The identification of cytokine signatures associated with early sensitization further suggests that biomarker-driven approaches may be useful for identifying individuals at risk and guiding early intervention strategies. This mechanistic continuum aligns closely with current clinical approaches in severe asthma, where monoclonal antibody therapies targeting IL-5 (e.g., mepolizumab, reslizumab, and benralizumab) and IL-4/IL-13 signaling (e.g., dupilumab) have demonstrated significant reductions in exacerbation rates, eosinophilic burden, and corticosteroid dependence (23).

Importantly, this finding suggests that the timing of intervention may be as critical as the therapeutic target itself. Anti-IL-5 therapies effectively mitigate established eosinophilic inflammation in patients with severe eosinophilic asthma. These findings raise the possibility that early modulation of IL-4-dependent pathways may influence later allergic outcomes. However, caution should be exercised when extrapolating findings from murine models to human infants, and further translational studies will be necessary to evaluate clinical relevance. Nonetheless, this study raises the possibility of a two-tiered therapeutic strategy: early-life interventions aimed at immune reprogramming and prevention, coupled with targeted biologic therapies for established disease phenotypes.

Furthermore, integration of immunologic profiling—including IL-4, IL-5, IgE, and eosinophil-related markers—may enable earlier identification of at-risk individuals and facilitate precision-guided interventions. Such strategies are particularly relevant given the heterogeneity of asthma endotypes and the variable response to biologic therapies across patient populations, especially those with severe eosinophilic asthma and hyper-eosinophilic syndrome (43, 44).

Collectively, these findings bridge fundamental immunologic mechanisms of early-life sensitization with established therapeutic strategies in severe eosinophilic asthma, highlighting a coherent and potentially transformative paradigm for disease prevention and treatment. These experimental data demonstrate that neonatal exposure to aeroallergens induces a dysregulated, exaggerated Th2 response in adulthood, driven in part by early IL-4–mediated immune programming. Notably, transient blockade of IL-4 during this critical neonatal window restores a more regulated immune phenotype, resulting in allergen responses comparable with those observed in non-sensitized adult mice. These findings provide compelling evidence that early-life cytokine signaling is not only permissive but instructive in shaping long-term immune trajectories.

Despite these promising implications, several limitations must be acknowledged. The use of OVA as a model antigen may not fully capture the complexity of natural environmental allergens. Additionally, while these data imply that IL-4 is a central driver of neonatal sensitization, further studies are needed to delineate the upstream cellular sources of IL-4, including potential contributions from ILC2 cells, basophils, and early activated T cells (45–47). Future investigations incorporating transcriptomic or epigenetic analyses would further clarify the mechanisms underlying long-term immune programming (37). Additionally, the safety, feasibility, and ethical considerations model of early-life cytokine-targeted interventions in humans require careful evaluation. Although IL-4 was directly manipulated experimentally, IL-5 was measured only as an outcome variable and was not functionally targeted; therefore, conclusions regarding IL-5 should be considered associative. The use of OVA in mouse models of allergic asthma is a well-established antigen system but may not fully reflect the complexity of naturally occurring environmental allergens. However, the use of inbred mice has long been the main model of choice to create a reproducible system for research studies to unravel the basic mechanisms of allergic diseases (48). Also, while the persistence of the observed phenotype into adulthood is consistent with long-term immune programming, alternative explanations such as sustained allergen-specific memory responses cannot be excluded. Finally, translation of findings from murine neonatal models to human infant immune development requires cautious interpretation.

In conclusion, this study demonstrates that neonatal exposure to aerosolized antigen promotes long-term allergic sensitization through IL-4-dependent mechanisms, with sustained effects on Th2 cytokine production, IgE responses, and eosinophilic inflammation. Therefore, these findings define a critical window in early immune development during which cytokine signaling pathways can shape lifelong immune outcomes and highlight IL-4 as a potential target for early intervention strategies aimed at preventing allergic disease. Thus, this work underscores the critical role of early-life Th2-biased immune responses in the pathogenesis of allergic disease and positions IL-4 and IL-5 as complementary therapeutic targets across the disease spectrum. By analyzing cytokine profiles, this study contributes to ongoing efforts to understand the neonatal response to allergens. The identification of reliable biomarkers has the potential to enhance treatment monitoring and clinical decision-making in allergic diseases (49–52). This may ultimately support more personalized and targeted therapeutic strategies, improving outcomes through individualized treatment. By linking neonatal immune programming with established biologic therapies, these findings support a paradigm shift toward earlier, mechanism-based intervention strategies that may ultimately reduce the global burden of asthma and allergic disease through prevention, personalization, and precision medicine.

## Data Availability

The original contributions presented in the study are included in the article/supplementary material. Further inquiries can be directed to the corresponding author.
